# Genomic Characterization of Pan‐Drug Resistant *Klebsiella pneumoniae* KPNW Isolated From UTI Patient in Bangladesh

**DOI:** 10.1002/mbo3.70064

**Published:** 2025-09-30

**Authors:** Md. Wahid Murad, G. M. Shah Poran, Kalpana Baidya, Nazmul Ahsan

**Affiliations:** ^1^ Department of Genetic Engineering and Biotechnology University of Dhaka Dhaka Bangladesh

**Keywords:** antibiotic resistance, antimicrobial resistance, genome sequence, *Klebsiella pneumoniae*, pan‐drug resistance, urinary tract infection

## Abstract

*Klebsiella pneumoniae* is a Gram‐negative opportunistic bacterial pathogen and a common cause of urinary tract infections (UTIs). In Bangladesh, a rapid increase in antibiotic resistance in *K. pneumoniae*, with a concerning prevalence of pan‐drug resistant (PDR) isolates, is severely limiting treatment options and posing a threat to public health. *K. pneumoniae* isolate KPNW was found in a clinical urine sample of a UTI patient from Dhaka, Bangladesh. In the disc diffusion test, KPNW showed resistance to 28 antibiotics across multiple classes, including carbapenems and colistin. Whole genome sequencing, assembly, and annotation yielded a 5.48 Mb genome encoding 5554 genes. Four plasmid replicons—lncFIB(K), lncFII(K), lncN2, and lncX3—were identified, all having known associations with antimicrobial resistance (AMR). KPNW belongs to multilocus sequence typing sequence type ST15 and capsular type KL112 and O1v2. We detected 42 AMR genes, including extended‐spectrum β‐lactamases, carbapenemase *bla*_NDM‐1_, and other families of AMR determinants, which altogether confer resistance to all clinically relevant antibiotics. We also identified 58 virulence determinants, including types 1 and 3 fimbrial proteins, enterobactin, and type VI secretion system proteins. However, major hypervirulence determinants were absent. Heavy metal resistance operons for arsenic, copper, and silver resistance were also detected. KPNW harbors multiple mobile genetic elements, some adjacent to AMR and virulence genes, indicating its capacity for horizontal gene acquisition and evolution as PDR, virulent, and heavy metal resistant. The findings of this study will have implications in public health and help better understand future trends of infections, plan effective treatment strategies, and surveillance.

## Graphical abstract

*Klebsiella pneumoniae* isolate KPNW showed resistance to all clinically relevant antibiotics. Upon whole genome sequencing and characterization, we detected a large number of antimicrobial resistance genes, resistance‐associated point mutations, virulence determinants, heavy metal resistance genes, and mobile genetic elements in errorits genome.

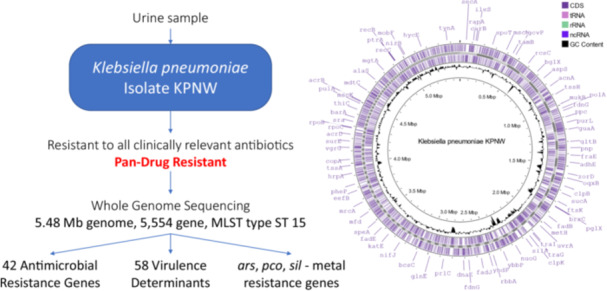

## 1 Introduction

*Klebsiella pneumoniae* is an encapsulated, facultatively anaerobic, Gram‐negative, nonmotile bacterium belonging to the family Enterobacteriaceae and order Enterobacterales. It can cause urinary tract infections (UTIs), sepsis, bacteraemia, meningitis, and pyogenic liver abscesses despite being initially isolated from pneumonia patients' lungs. Nowadays, *K. pneumoniae* is considered one of the most frequent pathogens causing nosocomial infections due to its high level of virulence and resistance to antibiotics (Chang et al. 2021; Paczosa and Mecsas 2016; Wyres et al. 2016). Other than *K. pneumoniae*, bacterial pathogens usually causing UTI include *Escherichia coli*, *Proteus mirabilis*, *Staphylococcus saprophyticus*, *Enterococcus faecalis*, and *Pseudomonas aeruginosa. K. pneumoniae* causes both community‐acquired and hospital‐acquired UTIs and often exhibits increased antibiotic resistance (Lin et al. 2010; Caneiras et al. 2019). Observation of increased levels of third‐generation cephalosporin resistance was reported in *K. pneumoniae* in the WHO Global Antimicrobial Resistance and Use Surveillance System Report 2022 (WHO 2022). Multidrug resistance and hypervirulence have become quite common in this opportunistic pathogen, which limits the treatment options (Cao et al. 2014; Moradigaravand et al. 2017; Bassetti et al. 2018; Tang et al. 2020).

In *K. pneumoniae*, various virulence factors are associated with human infection. The overproduction of capsule, the action of the plasmid‐encoded transcription factors, such as RmpA and/or RmpA2, siderophore (e.g., aerobactin), other iron transport systems (e.g., KfuABC), and so forth, make the *K. pneumoniae* hypervirulent (Russo and Marr 2019; Paczosa and Mecsas 2016; Wyres et al. 2020).

Over time, the use and misuse of antibiotics have led to the emergence and spread of strains with greater resistance. The differences between multidrug resistance, extensive drug resistance (XDR), and pan‐drug resistance are not very clearly defined. MDR is most frequently defined as resistant to three or more antimicrobial classes. Whereas XDR is commonly described as resistant to most standard antimicrobial regimens. Pan‐drug resistant (PDR) is usually defined as resistant to almost all antimicrobials commercially available or routinely tested. Magiorakos et al proposed interim standard definitions for these terms of acquired resistance in bacteria. They proposed enterobacteria to be PDR; it has to be nonsusceptible to all antimicrobials in their criteria list (Magiorakos et al. 2012). There have been reports of carbapenem‐resistant *K. pneumoniae* strains that are resistant to all commercially available antibiotics (Zowawi et al. 2015; Chen and Report 2017), known as PDR, making effective treatment impossible.

The emergence of multidrug and pan‐drug resistance is a common phenomenon driven by various ecological factors, such as long‐term antibiotic exposure, genome plasticity, and plasmid tolerance. This process involves the uptake of plasmids and other mobile genetic elements (MGEs) containing numerous antimicrobial resistance genes (ARGs) and the ongoing selection of mutants that carry these resistance genes.

This study reports the whole genome sequence of *K. pneumoniae* isolate KPNW. Isolated from the urine sample of a UTI patient, this bacterium was found to be resistant to all routinely tested antibiotics. We explored the genome of this PDR organism with the aim of identifying its genes responsible for antimicrobial resistance (AMR) and virulence, and associating these genes with any MGE present. To effectively combat PDR *K. pneumoniae*, understanding the molecular components of resistance mechanisms and identifying new pharmacological targets is crucial.

## 2 Materials and Methods

### 2.1 Sample Collection and Antibiotic Susceptibility Test

The bacterial isolate was collected from a local diagnostic center in Dhaka, Bangladesh, which was initially isolated from the urine sample of a patient with UTI. For isolation, urine was directly cultured on MacConkey (Oxoid Ltd., UK) agar plates by streaking 1 µL of the sample using a sterile inoculating loop (Karah et al. 2020). Colonies were isolated after incubation for 24 h at 37°C. The antibiotic susceptibility test of the isolate was performed using the disc diffusion method (Ericsson et al. 1960). Large (150 mm diameter) Mueller–Hinton agar plates were used for the test, and a bacterial inoculum of roughly 1 × 10^8^ CFU/mL was applied to the surface of the plates. Commercially manufactured antibiotic paper discs (Oxoid Ltd., UK) with predetermined concentrations were placed on the inoculated agar plates. After incubation for 16 h at 37°C, the zones of growth inhibition surrounding each antibiotic disc were measured (mm). The Clinical and Laboratory Standards Institute (CLSI) published standards (M100, 30th edition) were used to interpret the zone diameters of each medication (CLSI 2020).

### 2.2 Whole Genome Sequencing

A liquid culture of the isolate was subjected to DNA extraction and purification by using a Agencourt AMPure XP Kit. The quality and quantity of the extracted DNA were measured using a NanoDrop spectrophotometer (Desjardins and Conklin 2010). Adapter ligation and library preparation of the DNA fragments were done by the Ion Xpress Plus Fragment Library kit and the Ion Xpress Barcode Adapters Kit. Upon purification of the adapter‐ligated and nick‐repaired DNA by Agencourt AMPure XP Reagent and size selection of Library with the E‐Gel SizeSelect II Agarose Gel, whole genome sequencing was performed on an Ion GeneStudio S5 Series System, which is a semiconductor‐based NGS platform. This sequencing platform was chosen because it was available and accessible.

### 2.3 Genome Assembly and Annotation

The single‐ended reads produced by the sequencing platform were assembled using SPAdes version 3.15.4 (Bankevich et al. 2012). Contigs with bases longer than 1000 were selected for further analysis (Prime 2019). The quality of the assembly was assessed using QUAST version 5.2.0, a quality assessment tool for genome assemblies (Gurevich et al. 2013). The assembled genome was annotated using the NCBI Prokaryotic Genome Annotation Pipeline (PGAP) version 6.6 (Tatusova et al. 2016; Haft et al. 2018; Li, O'neill, et al. 2021). Genome maps were produced using Proksee (accessed on September 16, 2024), which uses CGView as its genome drawing engine (Grant et al. 2023; Stothard et al. 2019). This Whole Genome Shotgun project has been deposited at DDBJ/ENA/GenBank under the accession JAWJYM01. The version described in this paper is version JAWJYM010000000.1. The study data can be accessed at NCBI BioProject with accession PRJNA1029536 and BioSample SAMN37872047. The raw sequencing reads are deposited in NCBI SRA with accession SRR34563343, and the genome assembly can be accessed using GenBank accession GCA_041928875.1.

Strain identification of the genome sequence was done using StrainSeeker version 1.5.1_2 (Roosaare et al. 2017). The assembled contigs were aligned to the reference genome using the CLC Genome Finishing Module in the CLC Genomics Workbench version 22.0.1 (Matvienko 2015). The contigs were characterized as chromosomal or plasmid‐derived using three separate tools: RFPlasmid version 0.0.18 (Van Der Graaf‐Van Bloois et al. 2021), mlplasmids version 2.1.0 (Arredondo‐Alonso et al. 2018), and MobSuite version 3.1.9 (Robertson and Nash 2018) in Solu genomics platform version 1.0.278 (Saratto et al. 2025). Plasmid replicons were identified using PlasmidFinder version 2.1 (Carattoli et al. 2014; Camacho et al. 2009) and MobSuite version 3.1.9 (Robertson and Nash 2018) in Solu genomics platform version 1.0.278 (Saratto et al. 2025).

The multilocus sequence typing (MLST) sequence type was determined from the whole genome sequence using BIGSdb platform version 1.42.4 (accessible at https://bigsdb.pasteur.fr/) of Institut Pasteur (Jolley et al. 2018). BIGSdb‐Pasteur is a bacterial strain taxonomy and nomenclature platform based on genomic data, which was developed at the University of Oxford. The capsular types, including both K type and O type, were determined by Kaptive version 2.0.7 (Wick et al. 2018; Wyres et al. 2016; Lam et al. 2022).

### 2.4 Identification of ARGs, Virulence Genes, Heavy Metal Resistance Genes, and MGEs

ARGs were detected in the assembled genome using multiple tools, including ABRicate version 1.0.1 (accessible at https://github.com/tseemann/abricate), the Resistance Gene Identifier (RGI) tool version 6.0.1 in the Comprehensive Antibiotic Resistance Database (CARD) version 3.2.5 (Mcarthur et al. 2013; Alcock et al. 2020), VRprofile2 version 2.0 (Wang et al. 2022), ResFinder version 4.1 (Bortolaia et al. 2020; Camacho et al. 2009), and BacWGSTdb version 2.0 (Ruan and Feng 2016; Y. Feng et al. 2021). The details of the ARGs, including their antibiotic classes and mechanisms of resistance, were obtained from CARD (Mcarthur et al. 2013). Point mutations associated with acquired AMR were detected using PointFinder version 3.1.1 (Zankari et al. 2017) and AmrFinderPlus version 3.11.20 (Feldgarden et al. 2021) in the Solu genomics platform version 1.0.278 (Saratto et al. 2025). The pathogenicity of the isolate was determined using PathogenFinder version 1.1 (Cosentino et al. 2013). The presence of virulence genes within the genome assembly was detected using BacWGSTdb version 2.0 (Ruan and Feng 2016; Y. Feng et al. 2021). Identification of hypervirulence determinants by hvK2 multiplex PCR was done in silico using BIGSdb version 1.42.4 (Jolley et al. 2018). The heavy metal resistance genes were also detected by BIGSdb (Jolley et al. 2018). MGEs were identified by VRprofile2 version 2.0 (Wang et al. 2022) and Mobile Element Finder version 1.0.3 (Johansson et al. 2021). Associations of the MGEs with AMR and virulence genes were detected by VRprofile2 version 2.0 (Wang et al. 2022).

### 2.5 Retrieval of *K. pneumoniae* ST15 Genotype Data and Comparison With KPNW

ARGs, point mutations related to AMR, and virulence genes of additional *K. pneumoniae* ST15 isolates were retrieved to compare with KPNW. ARGs and point mutations related to AMR of *K. pneumoniae* ST15 isolates were retrieved from NCBI Pathogen Detection Isolates Browser (Accessible at https://www.ncbi.nlm.nih.gov/pathogens/) using filters *K. pneumoniae* as organism group and ST15 as strain. ARGs and virulence genes of *K. pneumoniae* ST15 isolates were retrieved from BacWGSTdb version 2.0 (Ruan and Feng 2016; Y. Feng et al. 2021). The frequency of the genes was calculated using PivotTable option in Excel version 1808 in Microsoft Office Professional Plus 2019. Phylogenetic tree based on single‐nucleotide polymorphism (SNP) strategy showing the evolutionary relationship of KPNW with other ST15 isolates was constructed using BacWGSTdb version 2.0 (Ruan and Feng 2016; Y. Feng et al. 2021), and an image of the phylogenetic tree was prepared in MEGA version 12 (Kumar et al. 2024).

## 3 Results

### 3.1 *K. pneumoniae* Isolate KPNW Is Resistant to All Antimicrobials Routinely Tested

The *K. pneumoniae* isolate KPNW was isolated from the urine sample of a UTI patient at a local diagnostic center in Dhaka, Bangladesh. Upon antimicrobial susceptibility assay, the isolate showed resistance against 28 available antibiotics. The antibiotics in the assay included gentamicin, imipenem, chloramphenicol, azithromycin, erythromycin, cotrimoxazole, amikacin, ampicillin, ciprofloxacin, cloxacillin, ticarcillin + clavulanic acid, piperacillin + tazobactam, meropenem, ertapenem, cefuroxime, ceftriaxone, ceftazidime, cefoperazone/sulbactam, cefixime, cefepime, aztreonam, amoxiclav, nitrofurantoin, colistin, nalidixic acid, levofloxacin, tigecycline, and minocycline. The isolate was found resistant against all 28 routinely tested antibiotics (Table 1). Hence, we characterized the isolate KPNW as PDR.

**Table 1:** Antimicrobial resistance profile of *Klebsiella pneumoniae* isolate KPNW.

Antibiotic	Antibiotic class	Phenotype^a^
Gentamicin	Aminoglycoside	R
Amikacin	Aminoglycoside	R
Chloramphenicol	Chloramphenicol	R
Imipenem	β‐Lactam: Carbapenem	R
Ampicillin	β‐Lactam: Penicillin	R
Cloxacillin	β‐Lactam: Penicillin	R
Ticarcillin + clavulanic acid	β‐Lactam/β‐lactamase inhibitor	R
Piperacillin + tazobactam	β‐Lactam/β‐lactamase inhibitor	R
Meropenem	β‐Lactam: Carbapenem	R
Ertapenem	β‐Lactam: Carbapenem	R
Cefuroxime	β‐Lactam: Cephalosporin	R
Ceftriaxone	β‐Lactam: Cephalosporin	R
Ceftazidime	β‐Lactam: Cephalosporin	R
Cefoperazone/sulbactam	β‐Lactam/β‐lactamase inhibitor	R
Cefixime	β‐Lactam: Cephalosporin	R
Cefepime	β‐Lactam: Cephalosporin	R
Aztreonam	β‐Lactam: Monobactam	R
Amoxiclav	β‐Lactam/β‐lactamase inhibitor	R
Azithromycin	Macrolide	R
Erythromycin	Macrolide	R
Nitrofurantoin	Nitrofuran	R
Colistin	Polymyxin	R
Ciprofloxacin	Quinolone	R
Nalidixic acid	Quinolone	R
Levofloxacin	Quinolone	R
Cotrimoxazole	Trimethoprim/sulfonamide	R
Tigecycline	Glycylcycline	R
Minocycline	Tetracycline	R

^a^ Antibiotic resistance is designated as R.

### 3.2 Genomic Characterization of *K. pneumoniae* Isolate KPNW

**Figure 1:**
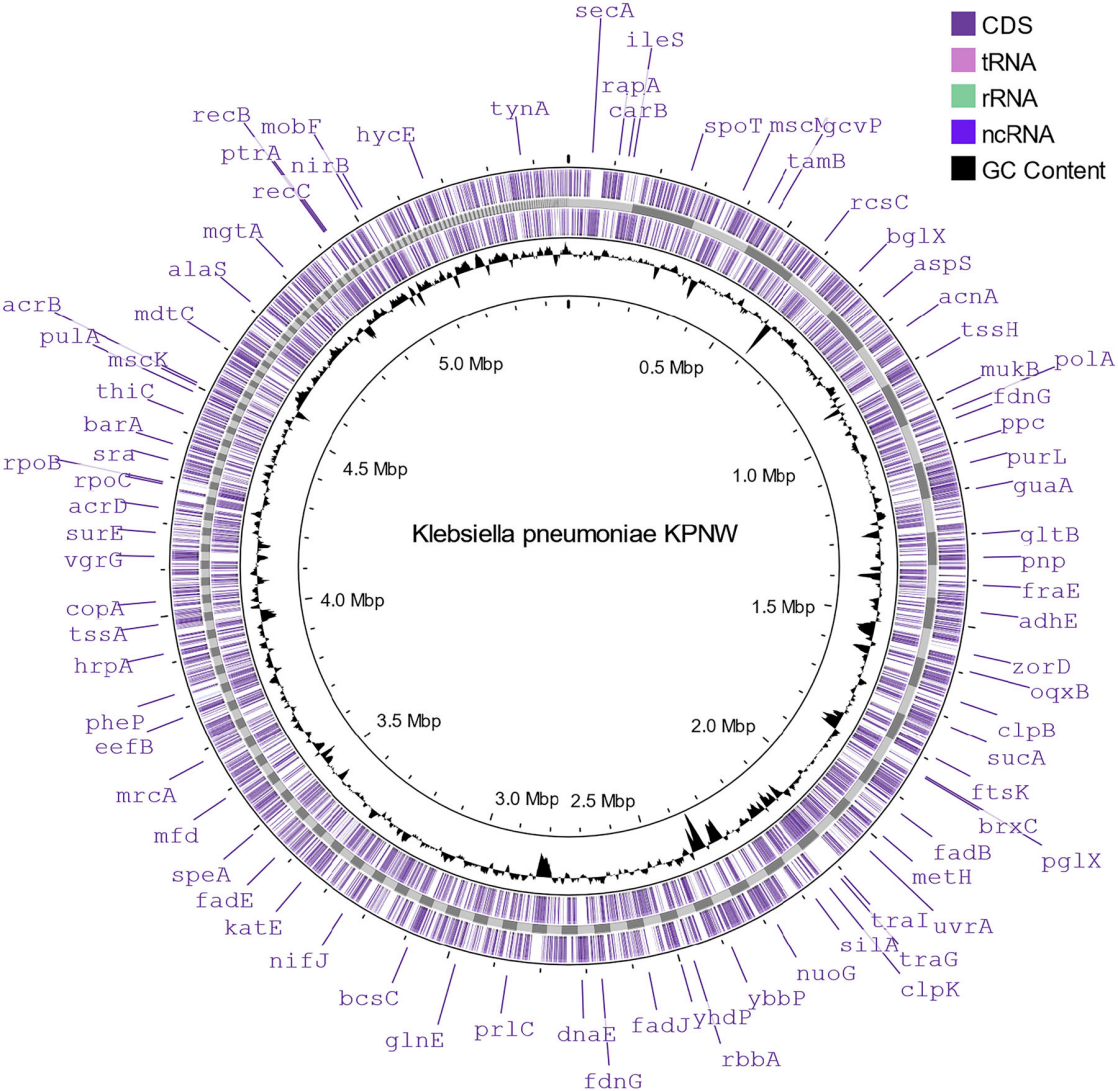
Genome map of *Klebsiella pneumoniae* isolate KPNW. The inner circle depicts GC content. The two outer circles depict two strands of the genome. ncRNA, noncoding RNA; rRNA, ribosomal RNA; tRNA, transfer RNA.

We sequenced and characterized its genome to identify which AMR and virulence genes the isolate carries. The whole genome was sequenced using the Ion GeneStudio S5 platform, which generated 1,241,166 single‐ended reads with 65.22× coverage. The reads were assembled de novo using SPAdes, which yielded 331 contigs. Using a cutoff value of 1000 bp 272 contigs were selected for further analyses. The total number of bases in the assembled sequence was 5,476,747 bp, with a guanine‐cytosine (GC) content of 57.09%. The sequence was annotated using the NCBI PGAP (Tatusova et al. 2016; Haft et al. 2018; Li, O'neill, et al. 2021), and it identified 5554 genes and 5492 coding DNA sequences along with 7 ribosomal RNAs, 47 transfer RNAs, and 8 noncoding RNAs (Table 2). Upon quality assessment of the de novo assembly with QUAST, the N50 and L50 values were predicted to be 38,869 and 38, respectively. The assembly was predicted to have a benchmarking universal single‐copy orthologs completeness score of 93.24%. A genome map of the assembly was produced using Proksee (Figure 1).

**Table 2:** Summary of assembly and annotation of the whole genome sequence of *Klebsiella pneumoniae* isolate KPNW.

Attribute	Value
No. of contigs	272
No. of bases	5,476,747
GC content (%)	57.09
Organism	*K. pneumoniae*
No. of genes	5554
No. of CDSs	5492
No. of rRNAs	4, 1, 2 (5S, 16S, 23S)
No. of tRNAs	47
ncRNAs	8
Pseudogenes	423
CRISPR arrays	1

Abbreviations: CDS, coding DNA sequence; CRISPR, clustered regularly interspaced short palindromic repeats; GC, guanine‐cytosine; ncRNA, noncoding RNA; rRNA, ribosomal RNA; tRNA, transfer RNA.

The sequence was identified to match with *K. pneumoniae* strain PMK1 with 100% relative frequency in the sample by StrainSeeker. The assembled contigs were further aligned to the reference genome of *K. pneumoniae* strain PMK1 (accession NZ_CP008929.1) using the CLC Genome Finishing Module in the CLC Genomics Workbench. Out of the total 272 contigs, 235 could be aligned with the PMK1 reference genome and joined to form a finished genome. A total of 37 contigs did not align with the PMK1 reference genome and hence could not be joined. After joining the contigs, the finished genome contained only four gaps greater than 10,000 bases with lengths 53,379, 101,250, 39,875, and 26,795 bp. The contigs were also classified as chromosomal or plasmid using three separate tools, RFPlasmid, mlplasmids, and MobSuite. We classified the contigs that did not align with the reference PMK1 genome and were called “Plasmid” by the tools to be plasmid‐derived for our isolate (31 Contigs—Contigs 24, 25, 40, 62, 81, 113, 153, 156, 162, 183, 185, 189, 190, 191, 194, 204, 212, 214, 218, 224, 227, 233, 234, 237, 243, 246, 248, 251, 253, 261, and 270). The rest of the contigs that did not align to the reference PMK1 genome and were called “Chromosomal” by the tools could be insertion sequences (IS) (Contigs 3, 23, 78, 209, 222, and 223).

PlasmidFinder identified four plasmid replicons in the genome by searching against its database of plasmid replicons from the order Enterobacterales. Identified plasmids were IncFIB(K) in contig 113, IncFII(K) in contig 189, IncN2 in contig 81, and IncX3 in contig 40 (Table 3). MobSuite further predicted the presence of at least three plasmids. Using assembled contigs, it reconstructed a 220,661 bp plasmid belonging to MOB cluster AA275 having replicon types IncFIB, IncFII, and rep_cluster_2183. The second one was 36,042 bp in length, belonged to AB161 MOB cluster, and carried IncN. These two plasmids were predicted as conjugative. The shortest one was 9338 bp long and had no origin of replication, and was nonmobilizable. MobSuite also detected but exluded a 36,227 bp long IncX3 replicon, addressing it as the presence of plasmid marker in chromosomal contig, possibly indicating an assembly error.

**Table 3:** Four plasmid replicons identified in the genome of *Klebsiella pneumoniae* isolate KPNW.

Plasmid	Identity (%)	Query/template length	Contig	Position in contig	Accession number
IncFIB(K)	98.75	560/560	113	9905..10464	JN233704
IncFII(K)	97.97	148/148	189	1613..1760	CP000648
IncN2	100	477/477	81	16036..16512	JF785549
IncX3	100	374/374	40	33934..34307	JN247852

The MLST sequence type of KPNW was determined as ST15 by BIGSdb. Capsular types were identified as KL112 for K type and O1v2 for O type by Kaptive.

### 3.3 KPNW Carries 42 ARGs

**Figure 2:**
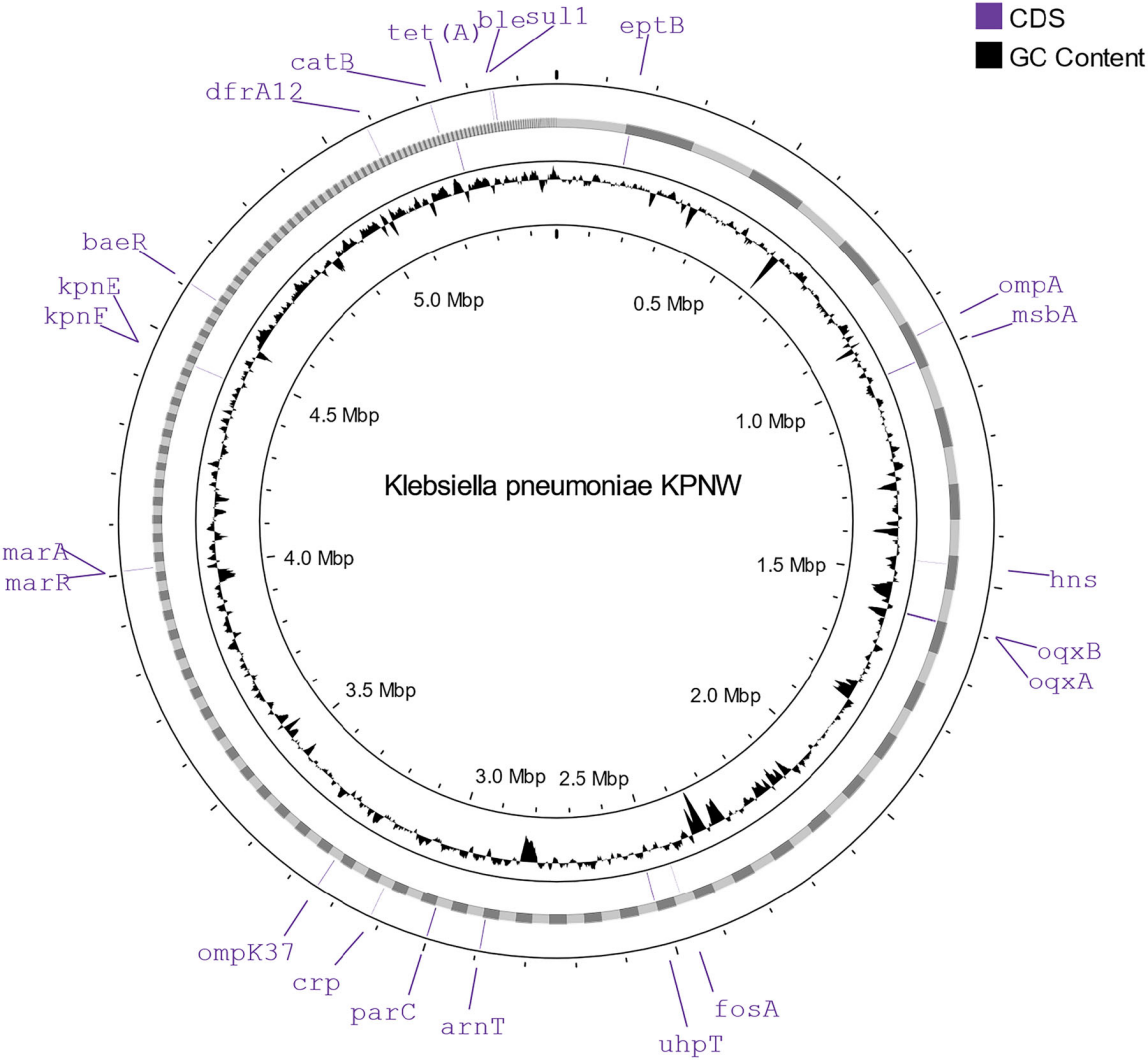
Genome map of *Klebsiella pneumoniae* isolate KPNW showing antimicrobial resistance genes.

We analyzed the presence of ARGs in the genome using ABRicate, the RGI tool in CARD, VRprofile2, ResFinder, and BacWGSTdb (Table 4). All the tools identified multiple ARGs at different locations (Figure 2). 42 ARGs were identified using these tools, where 17 genes were identified by more than one tool. Among these genes, 25 were identified in chromosomal contigs and 17 in plasmid‐derived contigs.

**Table 4:** Identified antimicrobial resistance genes, their locations, and the tools that identified the genes.

Gene	Contig	Origin	Start	End	ABRicate	RGI in CARD	VRprofile2	ResFinder	BacWGSTdb
*lptD*	1	C	115,021	117,369		■			
*pbp3*	1	C	67,557	69,323		■			
*eptB*	2	C	9007	10,680		■			
*msbA*	8	C	86,454	88,202		■			
*ompA*	8	C	36,014	37,084		■			
*h‐ns*	14	C	19,692	20,099		■			
*oqxA*	16	C	4076	5250	■	■	■	■	■
*oqxB*	16	C	900	4052	■		■	■	■
*fosA*	31	C	26,958	27,377	■	■	■	■	■
*uhpT*	33	C	1228	2619		■			
*arnT*	42	C	27,943	29,598		■			
*parC*	45	C	30,868	33,126		■			
*crp*	49	C	15,207	15,839		■			
*ompK37*	53	C	11,489	12,613		■			
*emrR*	70	C	18,397	18,927		■			
*kpnG*	70	C	19,053	20,225		■			
*kpnH*	70	C	20,241	21,779		■			
*bla*_SHV‐28_	77	C	12,301	13,161	■	■	■	■	■
*marA*	83	C	9382	9756		■			
*marR*	83	C	8927	9361		■			
*vanG*	93	C	13,594	14,724		■			
*kpnE*	107	C	12,017	12,379		■			
*kpnF*	107	C	11,701	12,030		■			
*baeR*	117	C	10,132	10,854		■			
*rsmA*	125	C	4950	5135		■			
*aadA1*	162	P	5111	5286	■				
*aadA2*	162	P	6188	6979	■	■	■	■	■
*bla*_TEM‐63_	162	P	49	265	■				
*dfrA12*	162	P	5283	5780	■	■	■	■	■
*catB1*	185	P	1672	2258	■				
*Rmtf*	185	P	3941	4720	■	■	■	■	■
*tet(A)*	191	P	1740	3014	■	■	■	■	■
*bla*_NDM‐1_	212	P	456	1268	■	■	■	■	■
*bleMBL*	212	P	1272	1637		■			
*qacE*	214	P	209	556		■	■	■	
*sul1*	214	P	550	1389	■	■	■	■	■
*mphA*	218	P	125	1045	■	■	■	■	■
*bla*_CTX‐M‐15_	224	P	1705	2580	■	■		■	■
*bla*_TEM‐104_	248	P	55	713	■			■	
*bla*_OXA‐1_	253	P	184	1014	■	■		■	■
*catB4*	253	P	1152	1647	■			■	
*aph(3′)‐Ia*	270	P	193	1008	■	■		■	■

Abbreviations: C = Chromosome, CARD, Comprehensive Antibiotic Resistance Database, P = Plasmid, RGI, Resistance Gene Identifier.

**Figure 3:**
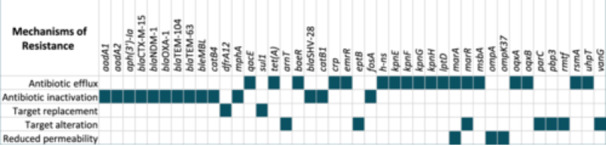
Mechanisms of antimicrobial resistance used by the detected genes.

The identified ARGs are found to confer their resistance by several mechanisms, including antibiotic inactivation, antibiotic target replacement, antibiotic efflux, antibiotic target alteration, and reduced permeability to antibiotics (Figure 3). Collectively, these genes mediate resistance to different classes of antibiotics (Table 5). *bla*_CTX‐M‐15_, *bla*_NDM‐1_, *bla*_OXA‐1_, *bla*_TEM‐104_, *bla*_TEM‐63_, *bla*_SHV‐28_, *crp*, *h‐ns, kpnG, kpnH, marA, marR, ompK37*, and *pbp3* confer resistance to penicillin, cephamycin, and penam antibiotics. *tet(A), h‐ns, kpnE, kpnF, marA, marR*, and *oqxA* cause resistance to tetracyclines. *bla*_CTX‐M‐15_, *bla*_NDM‐1_, *bla*_OXA‐1_, *bla*_TEM‐104_, *bla*_TEM‐63_, *bla*_SHV‐28_, *h‐ns, kpnE, kpnF, kpnG, kpnH, marA, marR, ompK37*, and *pbp3* are responsible for resistance to cephalosporins. One or more of the detected genes also confer resistance to other antibiotic classes that include fluoroquinolones, macrolides, sulfonamides, glycopeptide antibiotics, aminoglycosides, carbapenems, aminocoumarins, monobactams, rifamycin, diaminopyrimidine antibiotics, peptide antibiotics, phosphonic acid antibiotics, phenicol antibiotics, nitroimidazole, nitrofurans, disinfecting agents, and antiseptics.

**Table 5:** List of genes that provide resistance to different classes of antibiotics.

Antibiotic class	Antimicrobial resistance gene
Penicillin antibiotics	*bla*_CTX‐M‐15_, *bla*_NDM‐1_, *bla*_OXA‐1_, *bla*_TEM‐104_, *bla*_TEM‐63_, *bla*_SHV‐28_, *crp, h‐ns, kpnG, kpnH, marA, marR, ompK37, pbp3*
Tetracyclines	*tet(A), h‐ns, kpnE, kpnF, marA, marR, oqxA*
Cephalosporins	*bla*_CTX‐M‐15_, *bla*_NDM‐1_, *bla*_OXA‐1_, *bla*_TEM‐104_, *bla*_TEM‐63_, *bla*_SHV‐28_, *h‐ns, kpnE, kpnF, kpnG, kpnH, marA, marR, ompK37, pbp3*
Fluoroquinolones	*crp, emrR, h‐ns, kpnG, kpnH, marA, marR, oqxA, oqxB, parC, rsmA*
Macrolides	*mphA, crp, h‐ns, kpnE, kpnF, kpnG, kpnH*,
Sulfonamides	*sul1*
Glycopeptide antibiotics	*bleMBL, vanG*
Aminoglycosides	*aadA1, aadA2, aph(3′)‐Ia, baeR, kpnE, kpnF, kpnG, kpnH, rmtF*
Carbapenems	*bla*_NDM‐1_, *bla*_OXA‐1_, *bla*_TEM‐104_, *bla*_TEM‐63_, *bla*_SHV‐28_, *kpnG, kpnH, lptD, marA, ompK37*
Aminocoumarins	*baeR, lptD*
Monobactams	*bla*_TEM‐104_, *bla*_TEM‐63_, *marA, ompK37*
Rifamycin	*kpnE, kpnF, lptD, marA, marR*
Diaminopyrimidine antibiotic	*dfrA12, oqxA, rsmA*
Peptide antibiotics	*arnT, eptB, kpnE, kpnF, kpnG, kpnH, lptD, ompA*
Phosphonic acid antibiotics	*fosA, uhpT*
Phenicol antibiotics	*catB4, catB1, marA, marR, rsmA*
Nitroimidazole	*msbA*
Nitrofurans	*oqxA*
Disinfecting agents and antiseptics	*qacE, kpnE, kpnF, marA, marR*

Chromosomal point mutations may also be associated with acquired AMR. Such point mutations were detected in *acrR, ompK36, ompK37, ramR, gyrA, phoQ*, and *parC* genes using PointFinder and AmrFinderPlus (Table 6). The G385S mutation in *phoQ* causes resistance to colistin.

**Table 6:** Chromosomal point mutations associated with acquired antimicrobial resistance.

Gene	Amino acid change	Resistance
*Known mutations*		
*acrR*	P161R, G164A, F172S, R173G, L195V, F197I, K201M	Fluoroquinolone
*ompK36*	N49S, L59V, G189T, F198Y, F207Y, T222L, D223G, E232R, N304E	Cephalosporins
	A217S	Carbapenem
*ompK37*	I70M, I128M	Carbapenem
*ramR*	A19V	Tigecycline
*gyrA*	S83F, D87A	Ciprofloxacin
*phoQ*	G385S	Colistin
*parC*	S80I	Ciprofloxacin

### 3.4 KPNW Has 58 Virulence Determinant Genes

PathogenFinder was used to predict the probability of pathogenicity of the isolate. It matched the translated protein sequences of the isolate to its database of pathogenic and nonpathogenic families of organisms and found 305 matches to pathogenic families and 22 matches to nonpathogenic families with a minimum identity threshold of 100%. The calculated probability of the isolate being a human pathogen was 0.875, and it was predicted as a human pathogen.

**Figure 4:**
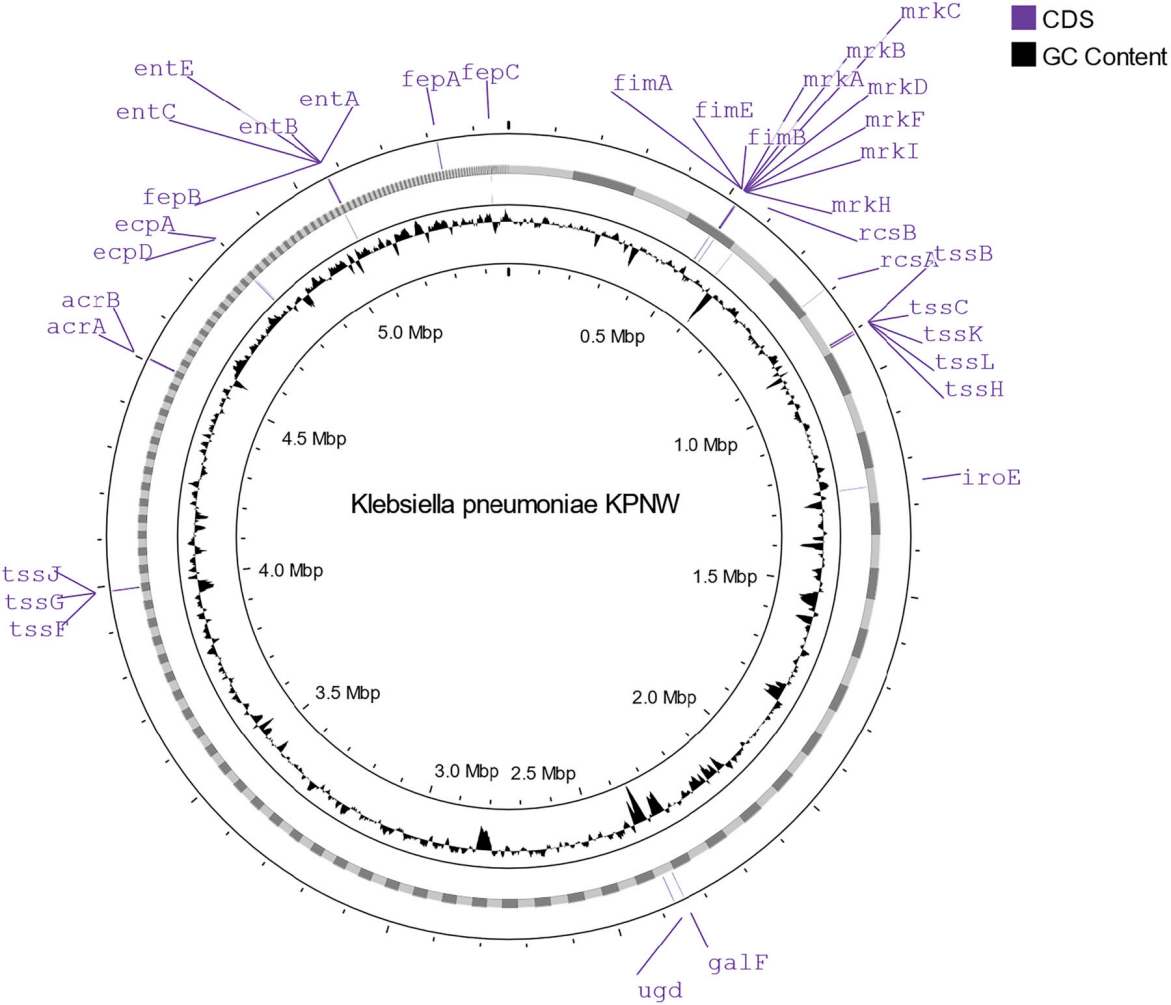
Genome map of *Klebsiella pneumoniae* isolate KPNW showing virulence genes.

Using another publicly accessible database called BacWGSTdb, we identified 58 virulence genes in the genome of the KPNW isolate (Table A1). The virulence genes are found at different locations throughout the genome (Figure 4).

In silico hvK2 multiplex PCR using BIGSdb yielded one exact match of 528 bp to locus KpI50233a allele 4 at contig 3 (3579–4106).

### 3.5 Operons for Resistance to Arsenic, Silver, and Copper Are Present in KPNW

**Figure 5:**
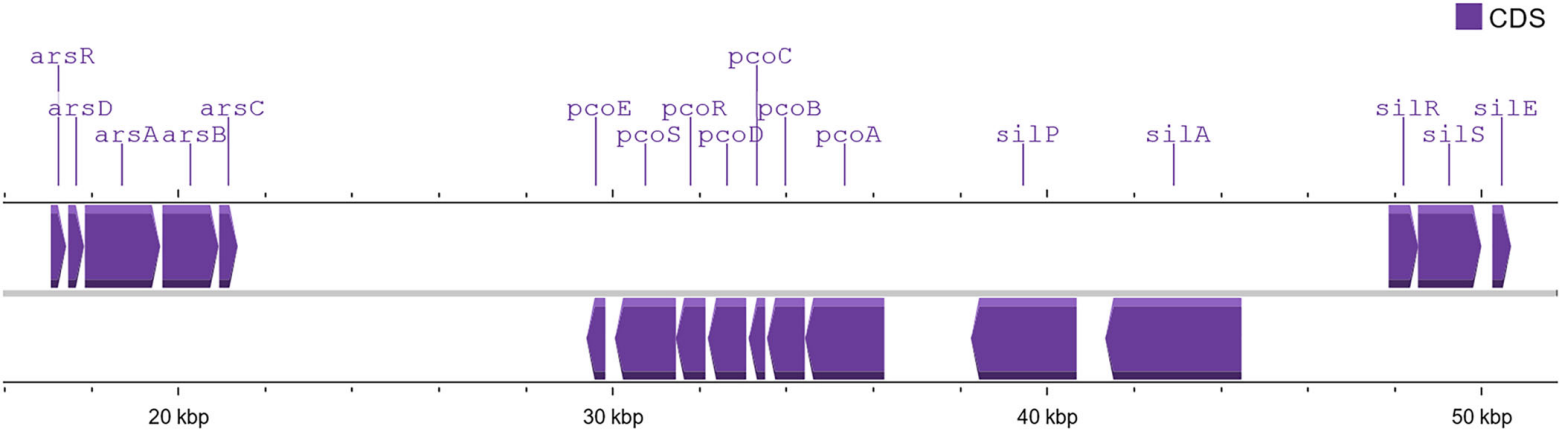
Relative locations of the metal resistance genes in contig 25.

Three metal‐specific resistance operons, *ars* for arsenic, *pco* for copper, and *sil* for silver, were detected in the KPNW genome by BIGSdb. All the arsenic, copper, and silver resistance genes were found in contig 25, which is a plasmid contig (Figure 5). Two additional copies of *silA* and *silR* were found in contig 123 (Table A2).

### 3.6 Association of ARGs and Virulence Genes With MGEs

MGEs like transposable elements, integron gene cassettes, and plasmids are crucial in the co‐selection of resistance. It was discovered that many of the first ARGs under investigation were carried on transposable elements. In this sequence, MGEs were detected using VRprofile2 and Mobile Element Finder. MGEs like ISs, transposons (Tn), prophages, and genomic islands are detected in 22 different contigs, including both plasmid and chromosome (Table 7).

**Table 7:** Detected mobile genetic elements, their types, features, and the tool they are detected with.

Contig	Origin	MGE type	Details	Position	Length	VRprofile2	MEF
3	C	Genomic_island	—	52461..62584	10,123	■	
5	C	Genomic_island	—	27604..39309	11,705	■	
13	C	Prophage	—	1484..37667	36,183	■	
15	C	Genomic_island	—	8832..41422	32,590	■	
15	C	IS/Tn	ISEc10	23767..24237	470	■	
15	C	IS/Tn	ISSgsp1	30998..31540	542	■	
15	C	IS/Tn	ISEhe3	44239..44496	257	■	
16	C	Prophage	—	5957..42909	36,952	■	
19	C	IS/Tn	ISEch12	45..311	266	■	
19	C	IScluster/Tn	ISEcl10/ISEcl10	6159..7090	931	■	
19	C	IS/Tn	IS903	24736..25659	923	■	■
24	P	IS/Tn	ISKpn14	130..405	275	■	
24	P	IS/Tn	ISKpn14	14627..14926	299	■	■
25	P	IScluster/Tn	ISPa52/ISKpn26/ISKpn14	117..2380	2263	■	■
25	P	IScluster/Tn	ISKpn38/ISKpn38/ISLad2	10058..12944	2886	■	
25	P	IScluster/Tn	ISEc38/ISEc38	27305..28925	1620	■	
25	P	IS/Tn	ISKpn25	50976..51380	404	■	
28	C	Genomic_island	—	339..38698	38,359	■	
29	C	Genomic_island	—	12262..41644	29,382	■	
58	C	IS/Tn	IS1R	29..304	275	■	
60	C	Genomic_island	—	8894..19958	11,064	■	
62	P	IS/Tn	ISKpn28	15458..16108	650	■	
62	P	IScluster/Tn	ISKpn33/ISKpn33	22225..23817	1592	■	■
81	P	IS/Tn	ISAba125	48..647	599	■	
86	C	Genomic_island	—	778..9263	8485	■	
91	C	Genomic_island	—	71..17978	17,907	■	
91	C	IScluster/Tn	ISEch12/ISSfl7	71..1439	1368	■	
138	C	IScluster/Tn	ISSod4/ISEch12	6470..10739	4269	■	
156	P	IS/Tn	IS903	126..335	209	■	
162	P	IScluster/Tn	TnpA_TnEcO26/TnpR_TnShes11	376..6979	6603	■	
162	P	Integron	—	4125..6979	2854	■	
212	P	IScluster/Tn	ISSen4/ISSen4	456..3269	2813	■	■
224	P	IS/Tn	ISEc9	1..1656	1656		■
251	P	IS/Tn	ISEcl1	54..1389	1336		■

Abbreviations: C = Chromosome, IS = Insertion Sequence, MEF = Mobile Element Finder, MGE = mobile genetic element, P = Plasmid, Tn = Transposon.

Five ARGs overlapped with two MGE locations (Table 8). *dfrA12*, *aadA1*, and *aadA2* overlapped with an IS cluster of Tn carrying an integron at contig 162, which is plasmid‐derived. On the other hand, *bla*_NDM‐1_ and *bleMBL* overlapped with an IS cluster at contig 212, which is also plasmid‐derived. These AMR genes may be obtained as cargo genes by these MGEs. VRprofile2 predicted that several virulence genes possibly overlapped with a genomic island in chromosomal contig 29 (Table 8).

**Table 8:** Association of mobile genetic elements with antimicrobial resistance genes and virulence genes.

Contig (origin)	MGE type	Details (position in Contig)	Overlapped gene (position in Contig)
*Antimicrobial resistance genes*			
162 (P)	IScluster/Tn	TnpA_TnEcO26/TnpR_TnShes11 (376..6979)	*bla*_TEM‐63_ (49..265), *dfrA12* (5283..5780), *aadA1* (5111..5286), *aadA2* (6188..6979)
162 (P)	Integron	(4125..6979)	*dfrA12* (5283..5780), *aadA1* (5111..5286), *aadA2* (6188..6979)
212 (P)	IScluster/Tn	ISSen4/ISSen4 (456..3269)	*bla*_NDM‐1_ (456..1268), *bleMBL* (1272..1637)
224 (P)	IS/Tn	ISEc9 (1..1656)	*bla*_CTX‐M‐15_ (1705..1580)
*Virulence genes*			
29 (C)	Genomic island	(12262..41644)	*gnd (*24795..26201*), manC* (26444..27859), *manB* (27882..29252), *ugd* (29416..30582), *wzm* (33569..34348), *wzt* (34348..35088), *wbbM* (35104..36999), *glf* (37015..38169), *wbbN* (38166..39059), *wbbO* (39072..40117)

Abbreviations: C = Chromosome, IS = Insertion Sequence, P = Plasmid, Tn = Transposon.

### 3.7 Comparison of KPNW With Available ST15 Isolates

To assess whether KPNW harbors a similar pattern of AMR and virulence genes compared with other ST15 isolates, genotype data of publicly available ST15 isolates were obtained from NCBI Pathogen Detection Isolates Browser and BacWGSTdb. AMR genotype and point mutations associated with AMR of 94 ST15 isolates were retrieved from the NCBI Pathogen Detection Isolates Browser. From BacWGSTdb, the AMR and virulence genotype of 447 ST15 isolates could be retrieved.

ARGs and point mutations related to AMR of 94 *K. pneumoniae* ST15 isolates were retrieved from NCBI Pathogen Detection Isolates Browser (Accessible at https://www.ncbi.nlm.nih.gov/pathogens/) using filters *K. pneumoniae* as organism group and ST15 as strain. ARGs and virulence genes of 447 *K. pneumoniae* ST15 isolates were retrieved from BacWGSTdb version 2.0 (Ruan and Feng 2016; Y. Feng et al. 2021). Three AMR genes detected in KPNW are found at frequencies over 95% in other ST15 isolates – *oqxB* (100%), *oqxA* (9.8%), and *fosA* (97.4%) (Table 9). Many of the AMR genes of KPNW were relatively infrequent among ST15 which included *bla*_CTX‐M‐15_ (65.8%), *bla*_OXA‐1_ (45.3%), *sul1* (44.5%), *tet(A)* (37.2%), *aadA1* (31.2%), *bla*_SHV‐28_ (16.8%), *aadA2* (15.7%), *aph(3′)‐Ia* (14.8%), *dfrA12* (13.7%), *bla*_NDM‐1_ (5.4%), *rmtF* (4.1%), and *bleMBL* (1.1%). On the other hand, some of the AMR genes detected in KPNW by the tools we used were not included in the AMR genotypes of NCBI pathogen or BacWGSTdb, and hence, abundance could not be calculated. These genes included *arnT*, *baeR*, *bla*_TEM‐104_, *bla*_TEM‐63_, *catB1*, *catB4*, *crp*, *emrR*, *eptB*, *h‐ns*, *kpnE*, *kpnF*, *kpnG*, *kpnH*, *lptD*, *marA*, *marR*, *mphA*, *msbA*, *ompA*, *ompK37*, *parC*, *pbp3*, *qacE*, *rsmA*, *uhpT*, and *vanG*. NCBI Pathogen also presented point mutations that are associated with AMR. Among 97 samples, the frequencies of mutations present in KPNW were calculated as *gyrA*_D87A (97.9%), *gyrA*_S83F (97.9%), *parC*_S80I (100.0%), and *ramR*_A19V (89.4%). Two infrequent mutations, *gyrA*_S83I (2.1%) and *ramR*_D152Y (2.1%), were not present in KPNW.

**Table 9:** Frequencies of AMR and virulence genes in publicly available ST15 genomes.

Antimicrobial resistance genes	
Frequency in ST15 genomes (*n* = 541)	
Not detected in KPNW	Detected in KPNW
*bla*_SHV‐106_ (76.5%), *aph(6)‐Id* (55.5%), *bla*_TEM‐1B_ (51.9%), *dfrA14* (43.3%), *sul2* (42.0%), *aph(3″)‐Ib* (41.4%), *aac(6')‐Ib‐cr* (39.4%), *mph(A)* (26.2%), *aac(3)‐IIa* (24.0%), *aac(6')‐Ib* (20.9%), *qnrB1* (20.5%), *bla*_KPC‐2_ (17.9%), *emrD* (17.4%), *aac(3)‐IId* (15.3%), *bla*_TEM‐1_ (14.6%), *armA* (13.9%), *bla*_OXA‐48_ (13.3%), *mph*(E) (12.6%), *msr(E)* (11.8%), *catA1* (11.3%), *bla*_OXA_ (11.1%), *bla*_TEM_ (11.1%), *dfrA23* (10.7%), *bla*_OXA‐9_ (10.4%), *bla*_SHV‐12_ (10.4%), *bla*_SHV‐11_ (8.5%), *bla*_SHV‐55_ (7.9%), *tet(D)* (7.6%), *arr‐3* (7.2%), *aadA16* (6.8%), *dfrA27* (6.5%), *aac(6')‐Ib3* (6.1%), *floR* (5.7%), *ARR‐2* (5.5%), *catB3* (5.5%), *bla*_TEM‐1A_ (5.4%), *aac(6')‐Ib‐cr5* (5.0%), *qnrB4* (4.6%), *bla*_VIM‐4_ (4.4%), *qnrS1* (4.4%), *aac(6')‐Ib*‐Hangzhou (4.3%), *bla*_OXA‐232_ (4.3%), *bla*_KPC‐3_ (4.1%), *bla*_VEB‐5_ (3.9%), *cmlA1* (3.7%), *bla*_DHA‐1_ (3.5%), *catA2* (3.5%), *bla*_SHV_ (3.1%), *dfrA15* (2.8%), *dfrA30* (2.8%), *qnrB6* (2.6%), *bla*_LAP‐2_ (2.4%), *dfrA1* (2.4%), *aph(3′)‐VI* (2.0%), *aac(6')‐IIc* (1.8%), *bla*_OXA‐10_ (1.8%), *bla*_VIM‐1_ (1.7%), *dfrA16* (1.7%), *dfrA5* (1.7%), *aac(3)‐IV* (1.5%), *aac(6')‐Ib11* (1.5%), *aadA5* (1.5%), *bla*_CTX‐M‐55_ (1.5%), *dfrA17* (1.5%), *qepA1* (1.5%), *qnrB2* (1.5%), *rmtB* (1.5%), *aph(4)‐Ia* (1.3%), *bla*_TEM‐1C_ (1.3%), *rmtC* (1.3%), *sul3* (1.3%), *bla*_CTX‐M‐3_ (1.1%), *bla*_NDM‐4_ (1.1%), *cml* (0.9%), *fosA3* (0.9%), *bla*_CMY‐6_ (0.7%), *bla*_CTX‐M‐14_ (0.7%), *bla*_NDM‐5_ (0.7%), *bla*_SCO‐1_ (0.7%), *bla*_SHV‐244_ (0.7%), *catB2* (0.7%), *dfrA18* (0.7%), *dfrA50* (0.7%), *dfrB1* (0.7%), *mcr‐9*_1_NZ (0.7%), *qnrB52* (0.7%), *rmtB1* (0.7%), *tet(B)* (0.7%), *aac(3)‐IIe* (0.6%), *bla*_CTX‐M‐33_ (0.6%), *bla*_IMP‐1_ (0.6%), *bla*_OXA‐534_ (0.6%), *bla*_TEM‐1D_ (0.6%), *erm(B)* (0.6%), *qnrB19* (0.6%), *aac(3)‐Ia* (0.4%), *ant(2″)‐Ia* (0.4%), *aph(3′)‐IIa* (0.4%), *bla*_CMY‐16_ (0.4%), *bla*_CTX‐M_ (0.4%), *bla*_CTX‐M‐199_ (0.4%), *bla*_CTX‐M‐27_ (0.4%), *bla*_KPC_ (0.4%), *bla*_LEN3_ (0.4%), *bla*_SHV‐1_ (0.4%), *bla*_SHV‐2_ (0.4%), *bla*_TEM‐122_ (0.4%), *dfrA22* (0.4%), *ere(A)* (0.4%), *qnrB9* (0.4%), *rmtD* (0.4%), *aac(2')‐IIa* (0.2%), *aac(6')‐Il* (0.2%), *aph(3′)‐VIb* (0.2%), *arr* (0.2%), *bla*_CMY‐2_ (0.2%), *bla*_CMY‐44_ (0.2%), *bla*_CTX‐M‐2_ (0.2%), *bla*_CTX‐M‐9_ (0.2%), *bla*_DHA‐25_ (0.2%), *bla*_GES‐11_ (0.2%), *bla*_IMP‐8_ (0.2%), *bla*_OXA‐181_ (0.2%), *bla*_OXA‐245_ (0.2%), *cat*(pC194) (0.2%), *dfrA25* (0.2%), *dfrA26* (0.2%), *dfrA51* (0.2%), *dfrB3* (0.2%), *lnu(F)* (0.2%), *mcr‐1.1* (0.2%), *mcr‐3.1* (0.2%), *mcr‐3.21* (0.2%), *mcr‐8* (0.2%), *oqxA10* (0.2%), *oqxB19* (0.2%), *oqxB5* (0.2%), *qnrA1* (0.2%), *qnrS* (0.2%), *tet(G)* (0.2%)	*oqxB* (100.0%), *oqxA* (99.8%), *fosA* (97.4%), *bla*_CTX‐M‐15_ (65.8%), *bla*_OXA‐1_ (45.3%), *sul1* (44.5%), *tet(A)* (37.2%), *aadA1* (31.2%), *bla*_SHV‐28_ (16.8%), *aadA2* (15.7%), *aph(3′)‐Ia* (14.8%), *dfrA12* (13.7%), *bla*_NDM‐1_ (5.4%), *rmtF* (4.1%), *bleMBL* (1.1%),

Virulence genes	
Frequency in ST15 genomes (*n* = 541)	
Not detected in KPNW	Detected in KPNW
*fes* (99.8%), *fepD* (99.6%), *fepG* (99.3%), *ybdA* (98.9%), *entF* (98.7%), *irp2* (51.7%), *ybtA* (51.7%), *ybtP* (51.7%), *ybtQ* (51.7%), *ybtS* (51.7%), *fyuA* (51.5%), *irp1* (51.5%), *ybtE* (51.5%), *ybtT* (51.5%), *ybtU* (51.5%), *ybtX* (30.4%), *kfoC* (14.1%), *iucD* (12.8%), *iucA* (12.5%), *iutA* (12.5%), *iucB* (12.3%), *iucC* (12.3%), *rmpA2* (8.9%), *wza* (1.6%), *rmpA* (0.9%), *astA* (0.4%), *papB* (0.4%), *chuS* (0.2%), *chuX* (0.2%), *chuY* (0.2%), *mucE* (0.2%), *papI* (0.2%), *pcrG* (0.2%), *sfaB* (0.2%), *tli1* (0.2%), *wcaG* (0.2%), *wcaH* (0.2%), *wcaI* (0.2%), *wcaJ* (0.2%)	*acrA* (100.0%), *acrB* (100.0%), *rcsB* (100.0%), *yagW*/*ecpD* (100.0%), *yagY*/*ecpB* (100.0%), *yagZ*/*ecpA* (100.0%), *entC* (99.8%), *entE* (99.8%), *fepA* (99.8%), *fepB* (99.8%), *galF* (99.8%), *gnd* (99.8%), *ugd* (99.8%), *cpsACP* (99.6%), *entA* (99.6%), *entB* (99.6%), *fepC* (99.6%), *fimC* (99.6%), *fimG* (99.6%), *rcsA* (99.6%), *yagX/ecpC* (99.6%), *fimF* (99.3%), *fimI* (99.3%), *icmF/tssM* (99.3%), *sciN/tssJ* (99.3%), *tssG* (99.3%), *ykgK/ecpR* (99.3%), *fimA* (99.1%), *fimD* (99.1%), *fimH* (99.1%), *fimK* (99.1%), *tssF* (99.1%), *mrkB* (98.9%), *mrkC* (98.9%), *mrkF* (98.9%), *mrkI* (98.9%), *mrkJ* (98.9%), *wzi* (98.9%), *mrkD* (98.7%), *fimE* (98.4%), *wzt* (98.0%), *fimB* (97.8%), *glf* (97.8%), *wzm* (97.8%), *wbbM* (97.5%), *wbbN* (97.3%), *mrkA* (96.6%), *wbbO* (94.9%), *hcp/tssD* (92.4%), *vasE/tssK* (92.4%), *clpV/tssH* (92.2%), *dotU/tssL* (92.2%), *vipA/tssB* (92.2%), *iroE* (91.9%), *mrkH* (90.4%), *vipB/tssC* (89.3%), *manB* (86.4%), *manC* (85.5%)

Abbreviation: AMR, antimicrobial resistance.

All the virulence genes detected in KPNW were present among the 447 genotypes obtained from BacWGSTdb. And all of them had frequencies above 85%. However, some of the genes frequently found in ST15 isolates, like, *fes* (99.8%), *fepD* (99.6%), *fepG* (99.3%), *ybdA* (98.9%), and *entF* (98.7%), were not detected in KPNW (Table 9).

**Figure 6:**
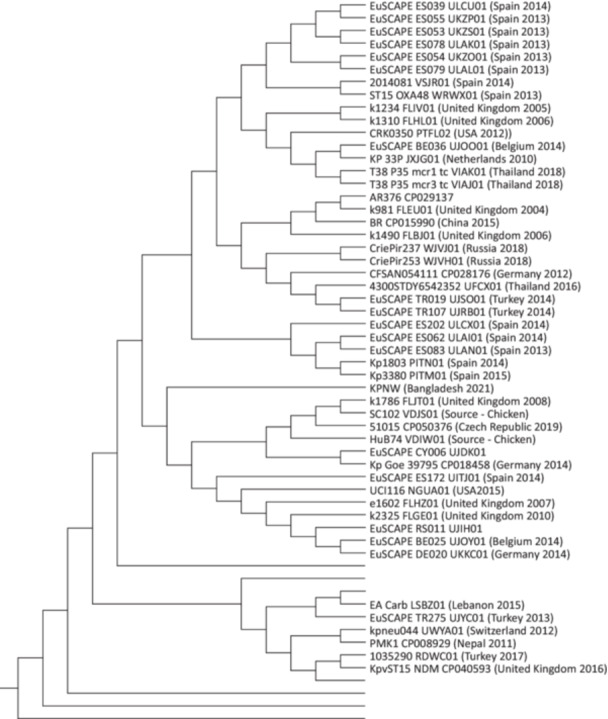
Phylogenetic tree showing the relationship of KPNW with other ST15 isolates. Only subtrees containing KPNW and PMK1 are shown here. The complete tree is available in Figure S1.

BacWGSTdb identified 192 isolates close to KPNW based on the SNP strategy and built a phylogenetic tree showing their relationship (Figure S1). In the phylogenetic tree, KPNW clustered together with isolates from Spain, the United Kingdom, the USA, Belgium, the Netherlands, Thailand, China, Russia, Germany, Turkey, and the Czech Republic, which were collected between 2004 and 2019 (Figure 6). PMK1 was placed in a neighboring subcluster.

## 4 Discussion

Multidrug resistance has been gradually increasing in Bangladesh over the last few years (Safain et al. 2020). A study from 2015 to 2019 showed that 80.69% of the clinical *K. pneumoniae* isolates (*n* = 42) were MDR. AMR genes like *bla*_CTX‐M_, *bla*_NDM‐1_, and *bla*_OXA_ were highly abundant in the MDR isolates of *K. pneumoniae* (Safain et al. 2020). Another study based on Chattogram division in Bangladesh reported 71.06% of the clinical *K. pneumoniae* isolates (*n* = 501) to be MDR in samples collected from two tertiary‐level hospitals, where the prevalence of MDR in *K. pneumoniae* isolated from urine samples was 67.56% (Tanni et al. 2022). The abundance of PDR strains of *K. pneumoniae* is increasing rapidly in Bangladesh. 14% of the isolates of *K. pneumoniae* analyzed (*n* = 145) from the intensive care unit of a tertiary care hospital in Dhaka from 2015 to 2017 were found to be PDR. These PDR isolates belonged to sequence types ST147, ST153, and ST14 (Okanda et al. 2021).

We found KPNW to be PDR and to harbor multiple virulence determinants and metal resistance genes. KPNW belongs to sequence type ST15 and capsular types KL112 and O1v2. ST15 *K. pneumoniae* has been globally associated with the production of extended‐spectrum β‐lactamases (ESBLs) and carbapenemases, causing notable hospital outbreaks (Lee et al. 2016). Previously, MDR ST15 *K. pneumoniae* has been reported from Bangladesh (Farzana et al. 2020; Hussain et al. 2023). However, there is still no report of PDR ST15 *K. pneumoniae* from Bangladesh. PDR isolates of ST15 have so far been reported in Brazil (Longo et al. 2019; De Araújo Longo et al. 2021) and England (Horváth et al. 2023). Hypervirulent XDR ST15 *K. pneumoniae* isolates were found in clinical specimens in Iran (Davoudabadi et al. 2022). Hypervirulent MDR strain of ST15 KL112 *K. pneumoniae* has been reported in China (Wu et al. 2023). The diversity of AMR determinants within ST15 clonal group isolates from different studies across the world indicates its high capacity for horizontal gene acquisition (Berglund et al. 2019).

KPNW carries at least 42 ARGs. Among these are several β‐lactamases, including *bla*_CTX‐M‐15_, *bla*_NDM‐1_, *bla*_OXA‐1_, *bla*_TEM‐104_, *bla*_TEM‐63_, and *bla*_SHV‐28_. The genes in *bla*_TEM_, *bla*_SHV,_ and *bla*_CTX‐M_ families are ESBLs, and *bla*_OXA_ and *bla*_NDM_ are well‐known carbapenemases (Castanheira et al. 2021; Nagshetty et al. 2021; Mojica et al. 2022). Although several members of the *bla*_OXA_ are observed to be ESBLs, the bla_OXA‐1_ found in KPNW is not an ESBL (Nagshetty et al. 2021). The New Delhi metallo‐β‐lactamase (NDM) genes are one of the most common metallo‐β‐lactamases isolated from clinical specimens. The metallo‐β‐lactamases can hydrolyze virtually all β‐lactam antibiotics with no clinically useful metallo‐β‐lactamase inhibitor. All these factors have made metallo‐β‐lactamases a global threat (Mojica et al. 2022).

There are several mechanisms by which these ARGs act. Among the 42 genes identified, the most common mechanism is antibiotic efflux, followed by antibiotic inactivation, target replacement, target alteration, and reduced permeability. KPNW showed resistance to all the antibiotics tested, including colistin. Colistin is an important antibiotic due to its use as the last resort for the treatment of carbapenem‐resistant enterobacteria. The primary genes responsible for colistin resistance are the mobile colistin resistance (*mcr*) genes (Elbediwi et al. 2019; Gharaibeh and Shatnawi 2019). KPNW does not have any *mcr* gene in its genome. A recent study (Sid Ahmed. 2024) reported the whole genome sequences of three PDR *K. pneumoniae* isolates from Qatar. These isolates, belonging to ST231 and ST383, also demonstrated resistance to colistin without the presence of any *mcr* gene. Other than this transferable mechanism, there are other intrinsic mechanisms for colistin resistance that have already been reported. Two genes, *arnT* and *eptA*, both present in KPNW, are reportedly associated with colistin resistance, where they remodel lipid A and neutralize its net negative charge by attachment of cationic sugar l‐Ara4N and phosphoethanolamine, respectively (Zhang et al. 2019). A two‐component EnvZ/OmpR‐mediated mechanism of colistin resistance has been demonstrated in *Aeromonas hydrophila*, where EnvZ/OmpR can upregulate the expression of *arnBCADTEF* operon, enhancing lipid A remodeling. Another two‐component system, PhoP/PhoQ, can mediate the addition of phosphoethanolamine to lipid A, conferring resistance to colistin in *A. hydrophila* (Liu et al. 2021). Both EnvZ/OmpR and PhoP/PhoQ systems are present in KPNW. Resistance to colistin in KPNW was intrinsic and predicted to be due to the presence of the G385S mutation in *phoQ*. KPNW is not only resistant to all antibiotic classes but also carries genes conferring resistance to common antiseptic agents. Thus, it may survive and spread easily in healthcare facilities, posing a greater threat of nosocomial infections.

We identified four plasmid replicons in the genome of KPNW. Two plasmids, lncFIB(K) and lncFII(K), are frequently found in *K. pneumoniae* and other enterobacteria to harbor many antimicrobial genes and virulence genes. These two plasmids were also observed in ST15 *K. pneumoniae* isolates in Japan (Soliman et al. 2021). The lncX3 plasmid was reported in *bla*_NDM‐1_ positive strains of *K. pneumoniae* in China (Li, Ding, et al. 2021). The lncN2 type plasmid was initially proposed from *E. coli* plasmid p271A. This plasmid carried *bla*_NDM‐1_ bracketed by two ISs ISSen4 and ISEc33 (Poirel et al. 2011). In KPNW, *bla*_NDM‐1_ is in a plasmid‐derived contig and adjacent to an ISSen4 IS, indicating its possible acquisition by horizontal transfer.

Simultaneous hypervirulence and pan‐drug resistance are very uncommon but aggressive in *K. pneumoniae*. Several factors determine hypervirulence. Hypervirulent strains usually contain *ybt*, *iuc*, and *iro* siderophore genes coding for yersiniabactin, aerobactin, and salmochelin, respectively, in addition to the core gene *ent* translating enterobactin. K1 and K2 are the predominant capsular types in hypervirulent strains of *K. pneumoniae* (Chen et al. 2023). Hypervirulent strains with other capsular types have also been observed, including KL112 (Wu et al. 2023). Hypermucoviscosity and colibactin production are also factors found in hypervirulent strains (Chen et al. 2023). In our study, KPNW contains at least 58 virulence factors. Notable virulence determinants are type 1 fimbrial proteins encoded by *fimABCDEFGHIK*, type 3 fimbrial proteins encoded by *mrkABCDFHIJ*, enterobactin genes *entABCE*, lipopolysaccharide‐associated genes *wzi*, *wzm*, *wzt*, type VI secretion system‐associated *tssBCDFGHJKLM* genes, and siderophore gene *iroE*. Type 1 fimbriae here is an essential virulence factor in UTI (Struve et al. 2009). However, KPNW lacks the presence of some major hypervirulence determinants, like, *iuc*, *iut*, *irp*, *fyu*, *clb*, *rmp*, and so forth. The presence of these markers is observed in ST15 isolates (Table 9). Among 447 isolates, the observed frequencies were *iucA* (12.5%), *iucB* (12.3%), *iucC* (12.3%), *iucD* (12.8%), *iutA* (12.5%), *irp1* (51.5%), *irp2* (51.7%), *fyuA* (51.5%), *rmpA2* (8.9%), and *rmpA* (0.9%). Multiplex PCR for identification of hypervirulent clones of *K. pneumoniae* serotype K2 was performed in silico, and only the KpI50233a locus was detected. KpI50233a is a marker for *K. pneumoniae sensu stricto*. No other loci in this assay came positive, which were markers of different hypervirulent clones of K2 strains (Bialek‐Davenet et al. 2014). Hence, KPNW is a virulent isolate, but we cannot call it hypervirulent.

Interestingly, we found arsenic, copper, and silver resistance operons in KPNW, and all in the same contig (Figure 5). Previously, co‐localization of antibiotic resistance genes and these heavy metal resistance genes was reported in *K. pneumoniae* from marine bivalves. Arsenic, copper, and silver resistance operons were located in the same plasmids, which had either lncFIB(K) or lncFII(K) replicons. These plasmids also carried ARGs, like, *bla*_SHV‐1_, *tet(D)*, *sul1*, *sul2*, *dfrA*, *bla*_TEM‐1_, *tet(A)*, and virulence factor *mrkABCDFJIH*. Simultaneous use of antibiotics and heavy metals as antifouling agents in aquaculture could facilitate the co‐selection of these plasmids (Håkonsholm et al. 2023). A similar scenario was observed in *K. pneumoniae* and *K. variicola* isolated from blood and cerebrospinal fluid samples of neonatal sepsis in England, where they found the co‐localization of *ars*, *sil*, and *pco* operons with antibiotic and virulence genes in lncFIB(K) or lncFII(K) plasmids of sizes ranging from 186 to 310 kb. The sustenance of such relatively large plasmids was speculated to confer virulence properties (Turton et al. 2022).

A bacterial strain identification tool, StrainSeeker, identified the isolate KPNW as the PMK1 strain of *K. pneumoniae*. PMK1 was first reported in Nepal in 2011 and was isolated from neonatal sepsis. PMK1 had a sequence type ST15 and carried four plasmids. The plasmids had lncHI1B/lncFIB, lncFII(K)/lncFIB(K), and lncFIB replicons. These plasmids contained a large number of antimicrobial and heavy metal resistance genes. PMK1 was susceptible to carbapenems and colistin (Stoesser et al. 2014). KPNW has the same clonal origin as PMK1 and common plasmid replicons. They also share AMR genes like *aadA2*, *armA*, *bla*_OXA‐1_, *bla*_CTX‐M‐15_, *bla*_SHV‐28_, and *bla*_NDM‐1_, and arsenic, copper, and silver resistance gene cassettes. KPNW likely evolved from the PMK1 strain and may have subsequently acquired additional resistance genes through horizontal gene transfer. Isolates close to KPNW were found in several countries and have been reported throughout the last two decades (Figure 6). It indicates that the prevalence of circulating ST15 clones and the gradual accumulation of resistance genes by horizontal transfer have led to the emergence of PDR isolates, like, KPNW. A recent population genomics study has reported that 69.2% of ST15 *K. pneumoniae* isolates (*n* = 287) were MDR and hypervirulent, with an observation of a sharp increase in the number of reports of such genomes from 2012 to 2021 in China (L. Feng et al. 2023). To clearly understand the extent of prevalence of such strains in South Asia or globally, genomics‐based surveillance systems are crucial along with routine phenotypic diagnostics. At least one study from Turkey reports successful treatment of PDR *K. pneumoniae* UTI in a 25‐day‐old neonate using a combination of ceftazidime and avibactum (Coskun and Atici 2020). With the increasing emergence of pan‐drug resistance, it is necessary to establish links between genomic findings and clinical decision‐making for the development of effective treatment guidelines.

The study has several limitations. Our assembled genome is fragmented, containing discrete contigs, which is due to the use of single‐ended short read‐based sequencing technology. The contigs were classified as chromosomal or plasmid‐derived, and plasmid replicons were identified. However, complete chromosome and plasmids could not be reconstructed. Finishing the chromosome and plasmid sequences could provide a better understanding of the genomic context of the identified resistance genes. Moreover, sequencing of more such PDR isolates is necessary to clearly understand the molecular epidemiology of the genes in the circulating PDR strains.

## 5 Conclusion

In this study, we present the assembled genome sequence of a PDR *K. pneumoniae* isolate called KPNW. KPNW was obtained from a urine sample and displays resistance to nearly all available classes of antibiotics, including carbapenems and colistin. We characterized its ARGs and virulence genes from the assembled genome. It carries multiple virulence factors but is not hypervirulent. KPNW also harbors three co‐localized heavy metal resistance operons for arsenic, copper, and silver. Several AMR and virulence genes were located adjacent to MGEs. The presence of four plasmid replicons and various MGEs indicates its high capacity for horizontal gene acquisition. KPNW possesses the capacity to spread rapidly both in community and hospital environments, posing a great threat to public health. Further research is needed to understand the significance of its resistance to both metals and antibiotics, uncover its underlying mechanisms, and develop effective treatment options.

## Author Contributions

**Md. Wahid Murad:** conceptualization (supporting), supervision (supporting), investigation, formal analysis (lead), writing – original draft (lead), writing – review and editing. **G. M. Shah Poran:** investigation, formal analysis, writing – original draft. **Kalpana Baidya:** investigation, formal analysis. **Nazmul Ahsan:** conceptualization (lead), funding acquisition, resources, supervision (lead), investigation, writing – review and editing.

## Ethics Statement

The authors have nothing to report.

## Conflicts of Interest

The authors declare no conflicts of interest.

## Supporting information


**Figure S1:** Complete phylogenetic tree showing the relationship of KPNW with 192 other available ST15 isolates.

**Supplementary File 1:** Raw results of analysis from bioinformatics tools.

## Data Availability

This Whole Genome Shotgun project has been deposited at DDBJ/ENA/GenBank under the accession JAWJYM01. The version described in this paper is version JAWJYM010000000.1. The study data can be accessed at NCBI BioProject with accession PRJNA1029536 and BioSample SAMN37872047. The raw sequencing reads are deposited in NCBI SRA with accession SRR34563343, and the genome assembly can be accessed using GenBank accession GCA_041928875.1. Raw results of analysis from bioinformatics tools are available in Tables S1–S18 (in Supporting Information File 1). The data that support the findings of this study are openly available in GenBank at https://www.ncbi.nlm.nih.gov/, reference number JAWJYM000000000.1.

## Appendix A

**Table A1:** Virulence genes with their locations and details. All the genes in this table are located in chromosomal contigs.

Gene	Contig	Similarity	Position	Details
*fimK*	4	99.19	61676..62917	Transcriptional regulator
*fimH*	4	99.56	63085..63993	Type 1 fimbrial adhesin precursor
*fimG*	4	99.4	64008..64508	Type 1 fimbrial minor component
*fimF*	4	98.87	64521..65051	Type 1 fimbrial minor component
*fimD*	4	99.62	65059..67702	Outer membrane usher protein
*fimC*	4	99.72	67751..68476	Periplasmic chaperone
*fimI*	4	99.53	68505..69140	Type 1 pilus biosynthesis fimbrial protein
*fimA*	4	99.64	69112..69660	Type 1 major fimbrial subunit precursor
*fimE*	4	99.18	70141..70749	Tyrosine recombinase
*fimB*	4	99.34	71214..71819	Tyrosine recombinase
*mrkA*	4	99.51	76484..77092	Type 3 fimbrial major pilin subunit MrkA
*mrkB*	4	99.72	77188..77889	Fimbrial chaperone protein MrkB precursor
*mrkC*	4	99.56	77901..80387	Fimbrial biogenesis outer membrane usher protein MrkC precursor
*mrkD*	4	100	80378..81373	Fimbrial adhesin protein precursor MrkD
*mrkF*	4	99.53	81387..82022	Type 3 fimbrial minor pilin subunit MrkF
*mrkJ*	4	99.86	82057..82773	Phosphodiesterase
*mrkI*	4	99.49	82917..83501	LuxR family regulatory protein
*mrkH*	4	100	83507..84217	Transcriptional activator
*rcsB*	5	99.85	10800..11450	Transcriptional regulator RcsB
*rcsA*	6	99.84	93326..93949	Transcriptional activator for ctr capsule biosynthesis
*vipA/tssB*	7	99.8	88585..89076	Type VI secretion system contractile sheath small subunit VipA
*vipB/tssC*	7	98.71	89119..90663	Type VI secretion system contractile sheath large subunit VipB
*vasE/tssK*	7	99.03	90673..92015	Type VI secretion system baseplate subunit TssK
*dotU/tssL*	7	99.42	92012..92701	Type VI secretion system protein, DotU/TssL family
*hcp/tssD*	7	98.98	94409..94900	Type VI secretion system protein, Hcp family
*clpV/tssH*	7	99.13	95164..97818	Type VI secretion system ATPase TssH
*iroE*	11	99.25	42600..43535	Siderophore esterase IroE
*galF*	29	98.77	6214..7110	UTP‐glucose‐1‐phosphate uridylyltransferase subunit GalF
*cpsACP*	29	94.75	7502..8130	Phosphatase phosphatidic acid phosphatase 2 family protein
*wzi*	29	87.52	9011..10525	Surface assembly of capsule
*gnd*	29	95.38	24795..26201	6‐Phosphogluconate dehydrogenase
*manC*	29	97.74	26444..27859	Mannose‐1‐phosphate guanylyltransferase
*manB*	29	98.61	27882..29252	Phosphomannomutase
*ugd*	29	96.57	29416..30582	UDP‐glucose 6‐dehydrogenase
*wzm*	29	99.23	33569..34348	Lipopolysaccharide O‐antigen ATP‐binding cassette (ABC) transport system transmembrane component
*wzt*	29	99.06	34348..35088	Lipopolysaccharide O‐antigen ABC transport system ATP‐binding component
*wbbM*	29	99.31	35104..36999	Glycosyltransferase
*glf*	29	98.7	37015..38169	UDP‐galactopyranose mutase
*wbbN*	29	98.77	38166..39059	Glycosyltransferase
*wbbO*	29	99.14	39072..40117	Glycosyltransferase family 1 protein
*icmF/tssM*	82	97.01	3452..6533	Type VI secretion protein TssM
*tssF*	82	99.43	9764..11518	Type VI secretion system baseplate subunit TssF
*tssG*	82	99.54	11482..12566	Type VI secretion system baseplate subunit TssG
*sciN/tssJ*	82	99.63	12544..13086	Type VI secretion system lipoprotein TssJ
*acrA*	110	99.33	8329..9522	Acriflavine resistance protein A
*acrB*	110	99.52	9545..12691	Acriflavine resistance protein B
*yagW/ecpD*	132	89.41	1173..2759	Polymerized tip adhesin of *Escherichia coli* common pilus (ECP) fibers
*yagX/ecpC*	132	87.64	2806..5312	*E. coli* common pilus usher EcpC
*yagY/ecpB*	132	88.89	5357..5977	*E. coli* common pilus chaperone EcpB
*yagZ/ecpA*	132	90.07	6088..6671	*E. coli* common pilus structural subunit EcpA
*ykgK/ecpR*	132	86.57	6746..7326	Regulator protein EcpR
*fepB*	160	99.58	104..1063	Iron‐enterobactin transporter periplasmic binding protein
*entC*	160	99.58	1240..2427	Isochorismate synthase
*entE*	160	99.38	2437..4044	Enterobactin synthase subunit E
*entB*	160	98.47	4058..4905	2,3‐Dihydro‐2,3‐dihydroxybenzoate synthetase, isochroismatase
*entA*	160	98.47	4879..5664	2,3‐Dihydroxybenzoate‐2,3‐dehydrogenase
*fepA*	206	97.79	1..1946	Outer membrane receptor FepA
*fepC*	244	99.37	876..1670	Iron‐enterobactin transporter ATP‐binding protein

**Table A2:** Metal resistance genes in the KPNW genome.

Gene	Length	Contig	Start position	End position	Origin^a^
*arsA*	1751	25	17836	19586	P
*arsB*	1290	25	19634	20923	P
*arsC*	1290	25	19634	20923	P
*arsD*	363	25	17456	17818	P
*arsR*	351	25	17056	17406	P
*pcoA*	1824	25	34431	36254	P
*pcoB*	900	25	33535	34434	P
*pcoC*	381	25	33115	33495	P
*pcoD*	930	25	32181	33110	P
*pcoE*	450	25	36483	36932	P
*pcoR*	687	25	31446	32132	P
*pcoS*	1401	25	30049	31449	P
*silA*	3147	25	41331	44477	P
*silA*	3147	123	1889	5035	C
*silE*	549	25	50132	50680	P
*silF*	354	25	45894	46247	P
*silG*	441	25	40804	41244	P
*silP*	2448	25	38230	40677	P
*silR*	681	25	47850	48530	P
*silR*	681	123	8274	8954	C
*silS*	1476	25	48523	49998	P

^a^ C = Chromosome; P = Plasmid.
