# Supplementary figures and images for: Genomic Characterization of Pan‐Drug Resistant Klebsiella pneumoniae KPNW Isolated From UTI Patient in Bangladesh
Source: Microbiologyopen. 2025 Sep 30;14(5):e70064. doi: 10.1002/mbo3.70064 (PMC12481437; doi:10.1002/mbo3.70064)

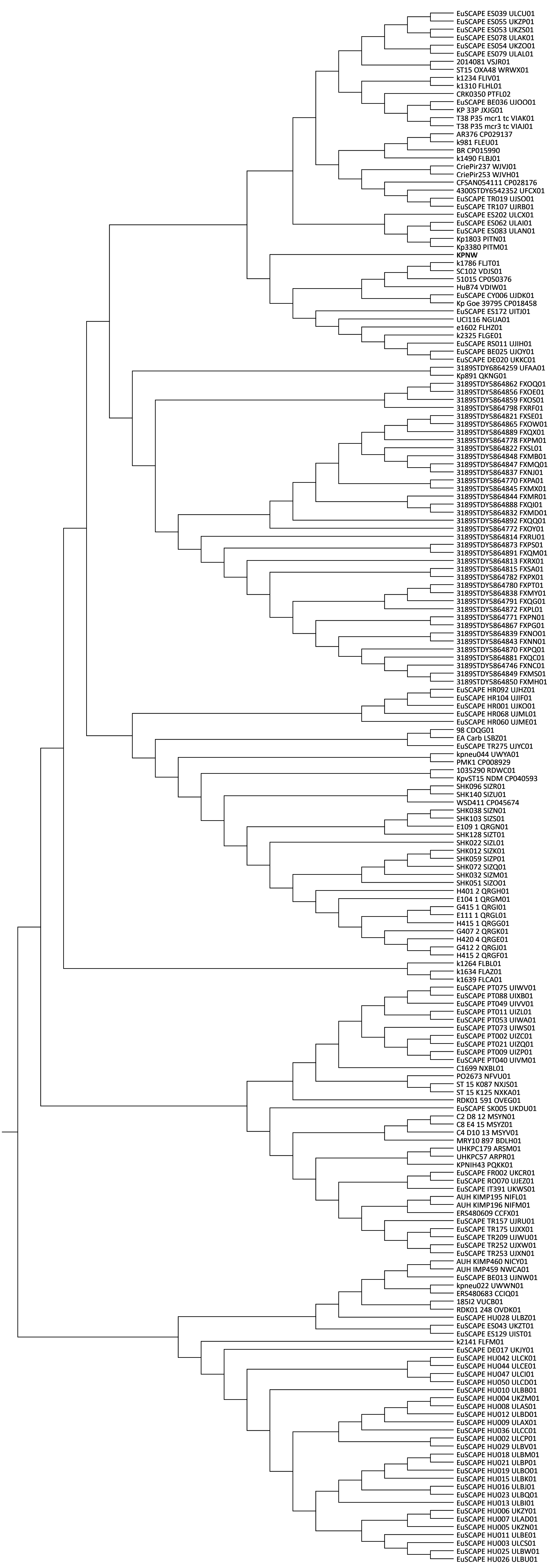

Supplement: Supplementary file 1 — Figure S1: Complete phylogenetic tree showing the relationship of KPNW with 192 other available ST15 isolates. [file MBO3-14-e70064-s002.pdf]
